# *In Situ* ATR-FTIR Analysis of Nalidixic Acid Degradation upon Oxygenation of Fe(II)-Sorbed Ferrihydrite and Maghemite Surfaces

**DOI:** 10.1021/acsomega.6c07545

**Published:** 2026-09-24

**Authors:** Mbolantenaina Rakotomalala Robinson, Ana Carolina Schuh Frantz, Alberto Mezzetti, Sylvie Nélieu, Xavier Carrier, Guillaume Morin

**Affiliations:** † Sorbonne Université, CNRS, Laboratoire de Réactivité de Surface, LRS, Paris F-75005, France; ‡ Sorbonne Université, CNRS, Laboratoire Interfaces et Systèmes Electrochimiques, LISE, Paris F-75005, France; § Institut de Minéralogie, de Physique des Matériaux et de Cosmochimie, IMPMC, UMR 7590, CNRS, Sorbonne Université, MNHN, 4 Place Jussieu, Paris Cedex 05 75252, France; ∥ Université Paris-Saclay, INRAE, AgroParisTech, UMR ECOSYS, Palaiseau 91120, France

## Abstract

The mechanisms of the abiotic degradation of antibiotics in redox-dynamic environments, such as aquifer sediments or hyporheic zones, remain poorly understood. This study investigates the transformation of nalidixic acid (NAL), a model quinolone antibiotic, triggered by the dark oxygenation of Fe­(II) sorbed onto two (oxyhydr)­oxides: nanomaghemite and ferrihydrite. Using *in situ* ATR-FTIR spectroscopy under continuous flow, we demonstrate that exposing the Fe­(II)-sorbed surfaces to dissolved O_2_ initiates the oxidation of adsorbed NAL over timescales of hours, without requiring light or added strong oxidants as H_2_O_2_. Real-time spectral analysis reveals mineral-dependent fates of transformation products. On Fe­(II)-sorbed maghemite, significant vibrational shifts indicate the oxidative cleavage of the pyridinic ring, mirroring mechanisms observed in heterogeneous Fenton systems, leading to the accumulation of adsorbed byproducts. Significant degradation of NAL is also observed upon oxygenation of Fe­(II)-sorbed ferrihydrite, with a slightly different end-product signal. These findings highlight that the aeration of Fe­(II)-bearing minerals is a potent natural pathway driving the deep structural transformation of recalcitrant contaminants during environmental redox cycles.

## Graphical abstract

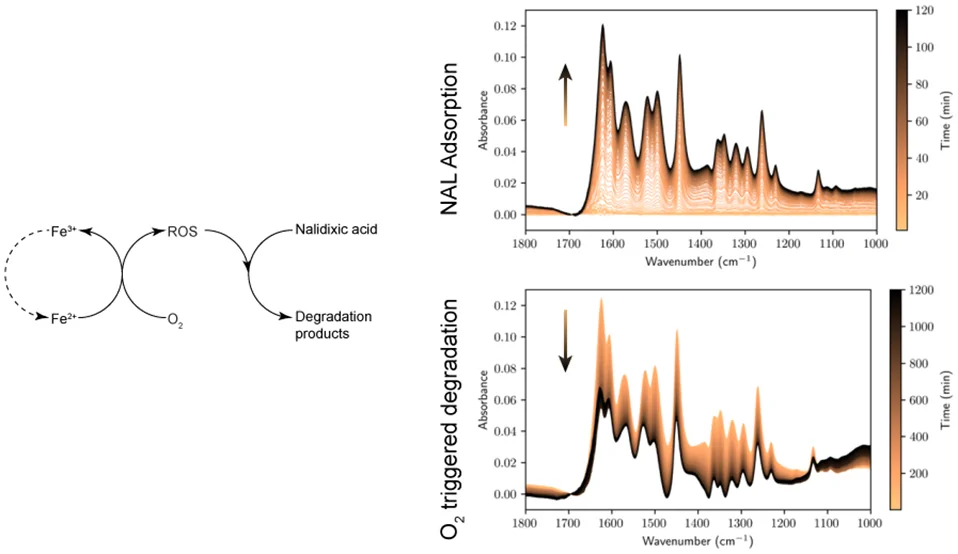

## Introduction

Intense research has been devoted to determining the role of natural Fenton-like processes in the ultimate fate of organic pollutants, since it has been reported that oxidative degradation can occur via oxygenation of Fe­(II)-bearing minerals^1–11^ as well as reduced soils^12,13^ and sediments.^14–18^ Indeed, it is now well recognized that reactive oxygen species (ROS), such as the hydroxyl radical •OH^6,8,9,12,13,15–17,19–21^ and possibly other reactive species, such as Fe­(IV),^22–28^ are produced when air-borne dioxygen reacts with such Fe­(II)-bearing substrates. Furthermore, several studies have reported subsequent oxidative degradation of a large variety of organic molecules via these heterogeneous Fenton-like reactions. These processes are considered to be of high importance for our understanding of the factors that govern the long-term fate of organic pollutants in the environment, since they are especially expected to occur during redox cycling of soils and sediments.^13,21,29–34^ Hydrological fluctuations in organic-rich porous media are typically known to favor dissimilatory iron reduction of Fe­(III) to Fe­(II) after oxygen consumption under water-saturated conditions, whereas reoxygenation of the Fe­(II)-bearing phases occurs in the vadose zone when the water table drops. Such redox cycles are thus investigated in the laboratory^35^ and in the field^14,21,32,34,36^ to evaluate ROS production and the degradation of organic pollutants.

Despite this bundle of evidence for the natural degradation of organic pollutants via dark oxygenation of Fe­(II)-bearing media, several questions remain about the mechanisms and kinetics of these reactions. For instance, the respective contributions of surface-bound and aqueous Fe­(II)^20,26,37^ and ROS^38,39^ are debated, which may have important implications for the effectiveness of these processes in the unsaturated or even dry vadose zone. In addition, while Fenton and photo-Fenton degradation pathways have been well depicted for several molecules, little is known about the degradation pathways of organic pollutants during dark oxygenation of synthetic and natural Fe­(II)-bearing substrates.

In this study, we aim to better understand the degradation pathway of a widely occurring pharmaceutical, nalidixic acid (NAL), at the solid–water interface under dark oxygenation of Fe­(II). We chose Fe­(II)-doped ferrihydrite as a substrate because it is relevant to the earliest stages of the dissimilatory reduction of poorly ordered iron oxyhydroxides in soils and sediments,^40–42^ and it is capable of O_2_-based Fenton-like degradation of organic molecules.^43^ We also compare the results with those obtained on another model substrate, Fe­(II)-sorbed maghemite, which has been reported as a suitable substrate for the Fenton degradation of NAL.^44^ We use Fourier-transform infrared spectroscopy in attenuated total reflectance mode (FTIR-ATR) to investigate the sorption of nalidixic acid onto Fe­(II)-sorbed maghemite and ferrihydrite, and we focus on its degradation after oxygenation under a continuous water flow.

## Materials and Methods

### Reagents

Nalidixic acid (NAL, C_12_H_12_N_2_O_3_), iron­(III) nitrate nonahydrate (Fe­(NO_3_)_3_·9H_2_O), potassium hydroxide (KOH), and iron­(II) chloride tetrahydrate (FeCl_2_·4H_2_O), all analytical grade, were purchased from Sigma-Aldrich and used without further purification. Absolute ethanol was obtained from VWR and used as received. Milli-Q water (18.2 MΩ·cm resistivity) was used for all experiments.

Fe­(II) stock solutions (0.02 M) were prepared by dissolving FeCl_2_·4H_2_O in deoxygenated Milli-Q water. For the antibiotic, separate nalidixate NAL^–^ stock solutions were prepared at concentrations of 2 ± 0.05 or 20 ± 0.05 mM. These were obtained by dissolving the required mass of nalidixic acid powder in deoxygenated Milli-Q water, followed by a pH adjustment to 9 (using 1 M NaOH) to ensure the dominance of the anionic species (pH > p*K*_a_) in the stock solution. The p*K*_a_ of nalidixic acid is reported to lie in the 5.8–6.2 range.^45^ Fe­(II) and NAL solutions were prepared and stored inside a glovebox under a nitrogen atmosphere (O_2_ < 10 ppm) to prevent early oxidation.

### Maghemite and Ferrihydrite Synthesis Procedures

Maghemite (Mh, γ-Fe_2_O_3_) was synthesized by thermal oxidation of nanomagnetite. Briefly, the magnetite precursor was prepared by coprecipitating Fe­(II) and Fe­(III) salts under alkaline conditions.^3,46^ The resulting black powder was then placed in an oven and heated at 200 °C for 18 h in air.^45^ During this process, the powder color changed from black to brown, indicating the transformation of magnetite into maghemite. Nitrogen gas sorption analysis indicated a Brunauer-Emmett-Teller (BET) specific surface area (SSA) of 49 m^2^·g^–1^.^45^ Rietveld analysis of the X-ray diffraction powder pattern yielded a mean coherent domain size of 12.7 ± 0.2 nm.^45^

Two-line ferrihydrite (Fh) was synthesized following the classic alkali precipitation method described by Schwertmann and Cornell.^47^ In brief, a 0.2 mol·L^–1^ ferric nitrate solution was slowly titrated to pH 7–8 using KOH under vigorous magnetic stirring and left to age overnight. The resulting reddish-brown gel was centrifuged at 6000 rpm for 30 min, then washed three times with Milli-Q water using successive centrifugation steps. The solid was finally vacuum-dried, and the recovered powder was ground manually in an agate mortar to obtain a fine, homogeneous material. The BET specific surface area was measured at 275 m^2^·g^–1^.

### Deposition of Fe­(II)-Sorbed Maghemite and Ferrihydrite onto the ATR Crystal

Fe­(II)-sorbed maghemite (Mh) or ferrihydrite (Fh) was deposited on the ZnSe ATR crystal from mineral suspensions prepared in the anoxic glovebox in a 1:1 mixture of deoxygenated Milli-Q water and absolute ethanol. Prior to deposition, an appropriate volume of the Fe­(II) stock solution was added to the mineral suspensions to reach Fe­(II) concentrations ranging from 0.18 to 11.1 mM, depending on the targeted surface loadings. To investigate the influence of the Fe­(II) surface density on nalidixic acid degradation, two experimental series were performed, as detailed below.

In the first series, 2 mg·mL^–1^ mineral suspensions were used. A volume of 1.2 mL of these suspensions was transferred into a polypropylene microcentrifuge tube, and 11 μL of the 0.02 M Fe­(II) stock solution was added to reach a Fe mass loading of 0.5 wt % with respect to Mh or Fh. Based on the specific surface areas, this corresponds to Fe­(II) surface densities of approximately 1.1 atoms·nm^–2^ for Mh and 0.2 atoms·nm^–2^ for Fh, referred to hereafter as the Mh@1.1 and Fh@0.2 experiments. While these densities differ due to the distinct specific surface areas of the minerals, this low-loading series was primarily intended to probe reactivity in a regime of isolated Fe­(II) sites.

In the second series, more concentrated mineral suspensions of 4.8 mg·mL^–1^ were used to target higher Fe­(II) surface coverages. For Mh, 1 mL of the suspension was mixed with 110 or 220 μL of the 0.02 M Fe­(II) solution to reach Fe loadings of 2.6 or 5.1 wt %, corresponding to surface densities of 5.6 and 11.3 atoms·nm^–2^, respectively, referred to hereafter as the Mh@5.6 and Mh@11.3 experiments. For Fh in this second series, the suspension volume was diminished to 0.5 mL, to ensure that the total mixture volume remained compatible with the ATR cell capacity (∼1.2 mL). Indeed, the high surface area of Fh required adding significantly larger volumes of Fe­(II) solution. Specifically, 309 and 618 μL were added to achieve Fe­(II) mass loadings of 14.4 and 28.8 wt %, corresponding to targeted Fe­(II) surface densities of 5.6 and 11.3 atoms·nm^–2^, respectively, referred to hereafter as the Fh@5.6 and Fh@11.3 experiments.

In all preparations, the mixtures were shaken vigorously and immediately deposited onto the ATR crystal. The whole suspension, including any remaining aqueous Fe­(II), was deposited and dried on the crystal. The Fe­(II) surface densities reported here are therefore nominal loadings, calculated from the amount of Fe­(II) added and the BET specific surface area of the mineral. Drying was carried out in a desiccator continuously purged with nitrogen within a glovebox to ensure a controlled atmosphere and to accelerate evaporation (complete drying was achieved in ∼2 h). Strict anoxic conditions were maintained throughout to minimize partial oxidation of Fe­(II). Once the deposit was fully dried, the flow-through ATR cell was hermetically sealed and connected to a closed-loop recirculation system. A volume of 15 mL of deoxygenated ultrapure water was circulated continuously for 4 h prior to spectral acquisition to stabilize the background signal and ensure system equilibration.

### ATR-FTIR Measurements

ATR-FTIR measurements were performed using a Bruker Vertex 80 spectrometer that was continuously purged with nitrogen and equipped with a mercury cadmium telluride (MCT) detector cooled with liquid nitrogen. Spectra were acquired in double-sided, forward–backward mode at a scanning speed of 160 kHz. The ATR crystal was a 10-reflection ZnSe crystal. The background spectrum (2000 scans) was acquired using the ZnSe crystal coated with the Fe­(II)-loaded Mh or Fh layer under continuous ultrapure water circulation. This background was recorded once before the adsorption step and was not renewed thereafter, so that any subsequent change in the optical coupling between the coating and the crystal appears as an apparent change in absorbance.

Immediately after background acquisition, the adsorption phase was initiated by injecting the NAL^–^ stock solution into the recirculating system. To accommodate the different mineral surface areas across the two experimental series, specific injection protocols were used. For the first series corresponding to the lowest Fe­(II) loadings, 0.2 mL of the 2 mM NAL^–^ stock was injected, resulting in initial NAL surface densities of approximately 2 molecules·nm^–2^ for the Mh@1.1 and 0.4 molecule·nm^–2^ for the Fh@0.2 experiments. For the second series with higher Fe­(II) loadings, i.e., the Mh@5.6, Mh@11.3, Fh@5.6, and Fh@11.3 experiments, the 20 mM NAL^–^ stock was used to target a comparable NAL density of approximately 5 molecules·nm^–2^ on both minerals. This was achieved by injecting 0.1 mL for maghemite films and 0.28 mL for ferrihydrite films. The nalidixate concentrations in the recirculating solution immediately after injection, before any sorption, were 0.026 mM for the Mh@1.1 and Fh@0.2 experiments, 0.132 mM for Mh@5.6 and Mh@11.3, and 0.366 mM for Fh@5.6 and Fh@11.3. The amounts introduced were chosen to match the targeted surface loadings, assuming complete sorption; as for the Fe­(II) loadings, the nalidixate surface densities quoted here are therefore nominal. Spectra were then acquired by collecting 500 scans every minute, with a spectral resolution of 4 cm^–1^.

After the adsorption period that lasted for 120 min, oxygen gas was bubbled into the mixing chamber at 5 mL·min^–1^ to initiate degradation of adsorbed NAL^–^. The latter reaction was monitored for 18 h under continuous O_2_ bubbling to ensure excess oxygen throughout. The recirculating circuit was not rinsed between the adsorption and the oxygenation steps: the same solution was kept in the loop throughout, so that the fraction of nalidixate not sorbed at the end of the adsorption step remained in the circulating solution, in equilibrium with the sorbed layer, during the oxygenation step. All sorption and degradation experiments were conducted under circumneutral pH conditions. Monitoring indicated that the pH remained within the 6.3–7.2 range throughout the experiments.

Data merging and plotting were performed by using Python. The full series of spectra are provided in the Supporting Information, whereas spectra merged over 4 min are presented in the figures of the main text.

## Results and Discussion

### Adsorption of Nalidixate onto the Fe­(II)-Sorbed Minerals

The temporal evolution of ATR-FTIR spectra of the nalidixate anion (NAL^–^) adsorbed onto Fe­(II)-sorbed mineral surfaces is shown in Figure 1. The data set encompasses six distinct systems covering a range of Fe­(II) surface densities: maghemite with 1.1, 5.6, and 11.3 atoms·nm^–2^ of sorbed Fe­(II) (panels a–c), and ferrihydrite with 0.2, 5.6, and 11.3 atoms·nm^–2^ of sorbed Fe­(II) (panels d–f). The growth of spectral bands confirms the progressive accumulation of NAL at the mineral-water interface for all studied conditions.

**Figure 1:**
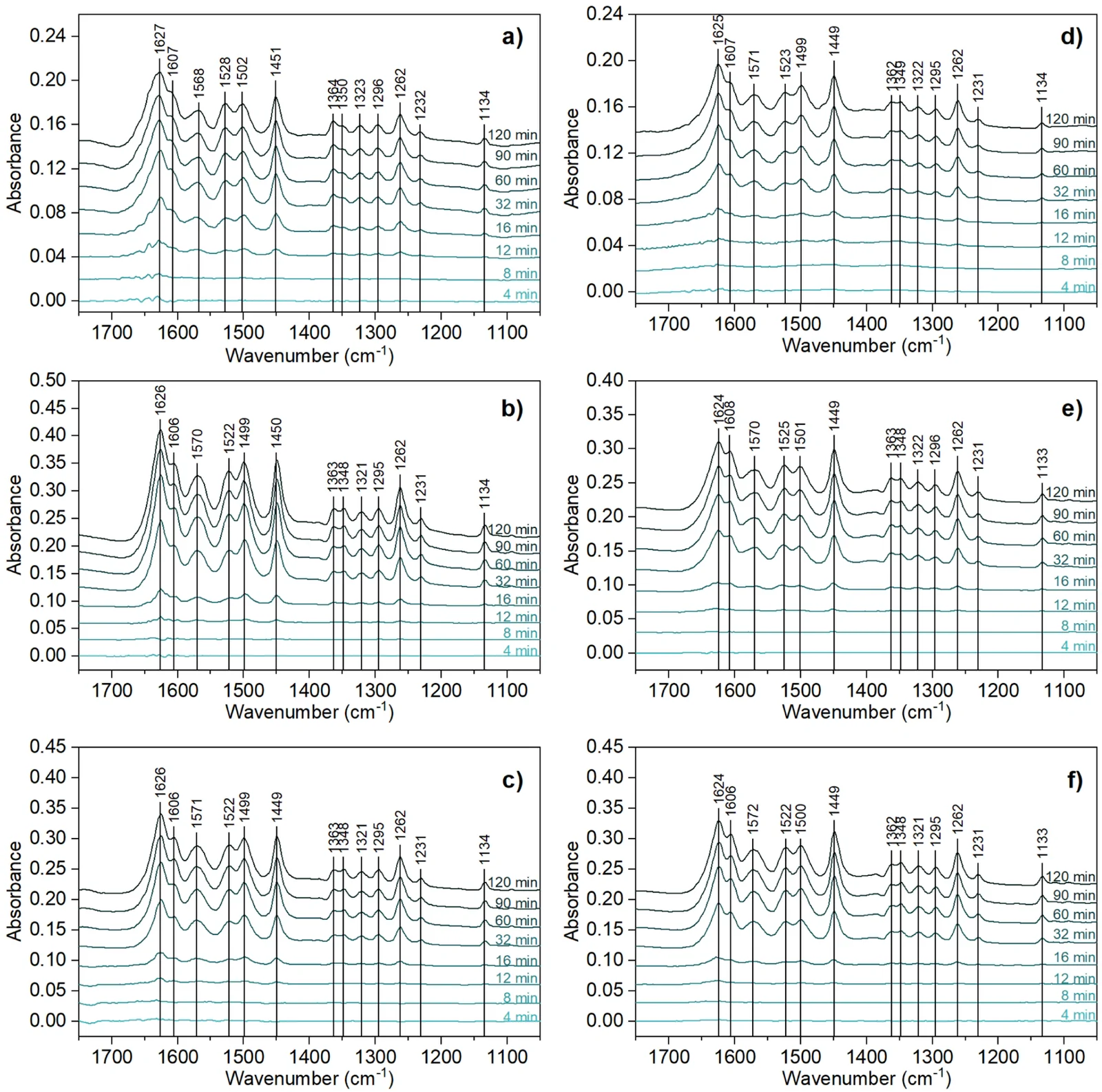
Time-series *in situ* ATR-FTIR spectra showing the adsorption of nalidixate onto Fe­(II)-sorbed mineral coatings. Panels (a–c) show maghemite, with sorbed Fe­(II) densities of a) 1.1, b) 5.6, and c) 11.3 atoms·nm^–2^ (Mh@1.1, Mh@5.6, and Mh@11.3, respectively). Panels (d–f) show ferrihydrite, with sorbed Fe­(II) densities of d) 0.2, e) 5.6, and f) 11.3 atoms·nm^–2^ (Fh@0.2, Fh@5.6, and Fh@11.3, respectively). Absolute band intensities are not directly comparable from one panel to another, as each spectrum is recorded against a background acquired on its own mineral coating.

In all samples, NAL adsorbs in its deprotonated form, as evidenced by the absence of the carboxylic acid C=O stretching band at 1710 cm^–1^ (Table 1). This is consistent with the pH of our experiments (6.3–7.2), which is higher than the NAL p*K*_a_ (5.8–6.2). The spectra are dominated by a strong band centered at 1626–1627 cm^–1^ for maghemite samples and 1624–1625 cm^–1^ for ferrihydrite samples. This band, accompanied by a shoulder around 1606–1608 cm^–1^, is assigned to the coupled stretching modes of the keto-carbonyl group and the asymmetric carboxylate (ν­(C=O)/ν­(COO^–^)_as_).^45^

**Table 1:** Characteristic ATR-FTIR Vibrational Bands (cm^–1^) Observed on Fe­(II)-Sorbed Maghemite (Mh) and Ferrihydrite (Fh) Coatings at the End of the Adsorption Period (t = 120 min) (Upper Line) and after 18 h of Dark Oxygenation (Lower Line)^a^

Mh@1.1	Mh@5.6	Mh@11.3	Fh@0.2	Fh@5.6	Fh@11.3	Vibrational mode assignment based on Schuh Frantz et al.^45^
1627	1626	1626	1625	1624	1624	νC=O carbonyl, νCOO^–^ as
1640	1632	1633	1625	1625	1625	
1607	1606	1606	1607	1608	1606	νC=O carbonyl, νCOO^–^ as
1611	1609	1610	1607	1608	1608	
1568	1570	1571	1571	1570	1572	νC–C 1st ring, νC–H
1564	1565	1565	1565	1567	1567	
1528	1522	1522	1523	1525	1522	νC–C and νC=N 2nd ring, δC–H
1531	1527	1528	1523	1527	1527	
1502	1499	1499	1499	1501	1500	νC–C and νC=N rings
1506	1503	1505	1498	1503	1503	
1451	1450	1449	1449	1449	1449	δC–H ethyl
1453	1451	1452	1450	1450	1450	
1262	1262	1262	1262	1262	1262	νCOO^–^ s, νC–C
1263	1263	1263	1262	1262	1262	

a Stretching modes are noted as ν and deformation modes as δ. Ring numbering refers to the molecular scheme displayed in Figure S7. Only the modes discussed in the text are listed; the complete band assignment of nalidixate is given in our previous study.^45^

Lower-frequency modes appear consistently for both Fh and Mh series: the band at 1568–1572 cm^–1^ is attributed to C–C stretching of the first ring; the band at 1522–1528 cm^–1^ corresponds to C–C/C=N stretching of the second ring; and the peak near 1499–1502 cm^–1^ involves skeletal ring vibrations.^45^ The symmetric carboxylate stretching mode (ν­(COO^–^)_s_) is observed at a fixed position of 1262 cm^–1^ for all six systems. These spectral signatures indicate that the coordination geometry of adsorbed nalidixate, likely a bidentate bridging or chelating mode,^45^ is similar for both mineral types (Mh vs Fh) and the Fe­(II) surface density (from 0.2 to 11.3 atoms·nm^–2^). These band positions are, however, close to those of aqueous nalidixate, so that they do not by themselves establish an inner-sphere coordination, as discussed below. The point of zero charge (PZC) of nanomaghemite particles of ∼10–15 nm in size, and obtained from nanomagnetite oxidation as in this study, typically ranges between 6.5 and 7.0.^48–53^ For two-line ferrihydrite synthesized from ferric nitrate^47^ as in this study, the PZC is reported to range between pH 7.8 and 8.7.^51,53,54^ At our working pH of 6.3–7.2, ferrihydrite therefore carries a net positive charge, whereas nanomaghemite is expected to exhibit a zero to slightly positive surface charge. Presorbed Fe­(II) is a cation and is thus expected to shift the surface charge of both coatings toward more positive values, so the above PZC values should be taken as a lower bound. Positive surface charges can favor electrostatic adsorption of deprotonated NAL but do not exclude inner-sphere coordination. Comparing the ATR-FTIR spectra of aqueous and sorbed NAL^–^ shows only minor shifts (Table S1). Hence, within the Nakamoto framework,^55^ the similarity in carboxylate stretching band positions, especially Δν­(COO^–^)_sorbed_ ≈ Δν­(COO^–^)_ionic_, is considered characteristic of a bridging bidentate coordination, as discussed in our previous study.^45^ This cannot, however, exclude the presence of outer-sphere anionic NAL^–^. On ferrihydrite, an unperturbed carboxylate combined with a positively charged surface at the working pH makes an outer-sphere contribution plausible. On maghemite, where the surface lies close to its point of zero charge and the perturbation is small but nonzero, an inner-sphere contribution is the more likely origin of the sorption observed.

### NAL Degradation: Spectroscopic Evidence of Ring Opening

Control experiments carried out with the same experimental setup, without sorbed Fe­(II), have been reported for both minerals, using H_2_O_2_ as the oxidant: no detectable transformation of adsorbed nalidixate was observed on maghemite^44^ or on ferrihydrite.^56^ These controls bound, a fortiori, the reactivity of Fe­(II)-free surfaces under the milder oxidizing conditions used here. Upon initiating oxygenation, the evolution of peak positions reveals a striking difference in reactivity between the maghemite and ferrihydrite systems, providing direct insight into the degradation pathways (see Figure 2 and Table 1).

**Figure 2:**
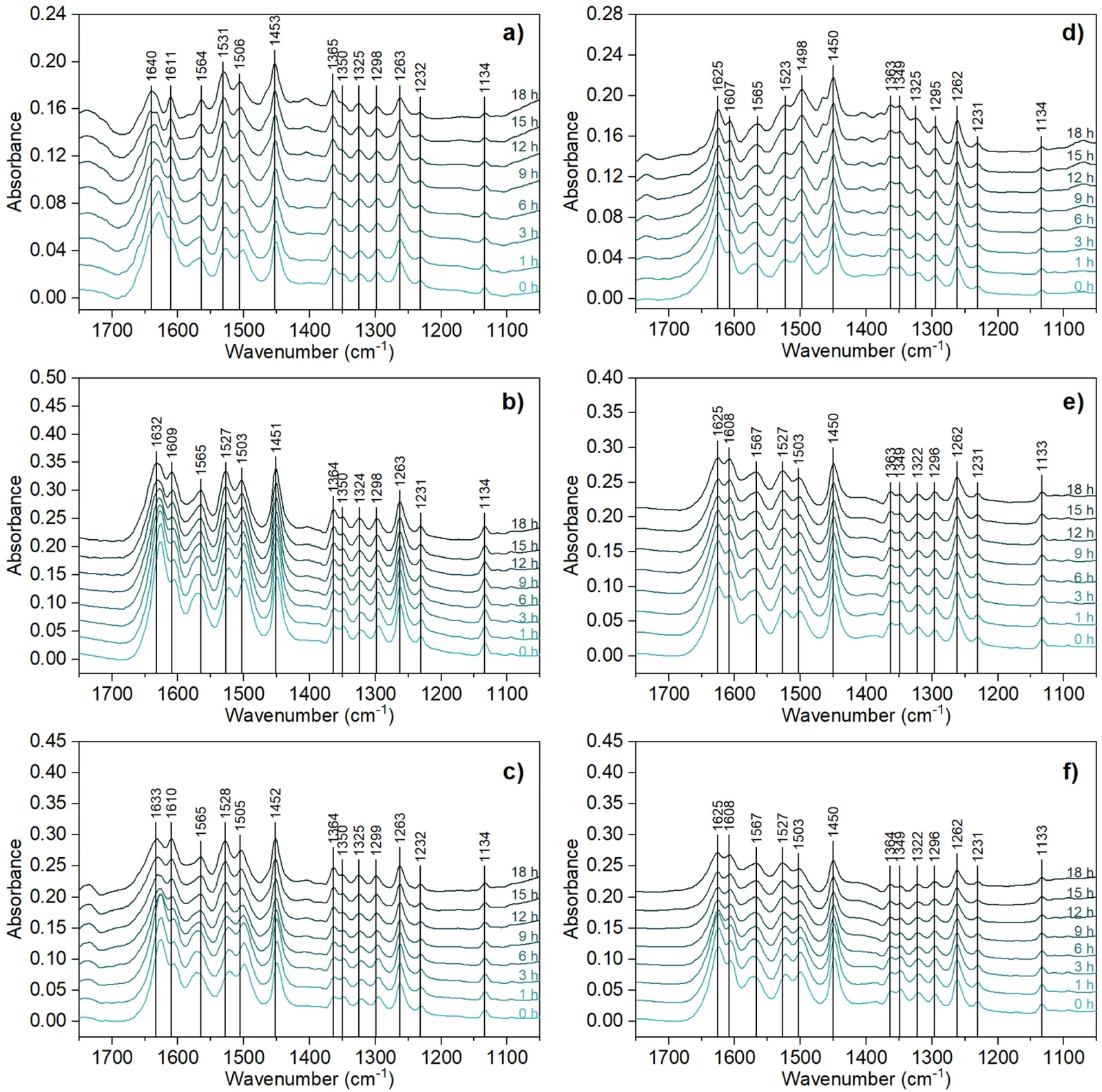
Temporal evolution of *in situ* ATR-FTIR spectra during the dark oxygenation of preadsorbed NAL on Fe­(II)-sorbed minerals. Panels (a–c): maghemite series with Fe­(II) densities of a) 1.1, b) 5.6, and c) 11.3 atoms·nm^–2^ (Mh@1.1, Mh@5.6, and Mh@11.3, respectively). Panels (d–f): ferrihydrite series with Fe­(II) densities of d) 0.2, e) 5.6, and f) 11.3 atoms·nm^–2^ (Fh@0.2, Fh@5.6, and Fh@11.3, respectively).

For the maghemite series (Mh@1.1, Mh@5.6, and Mh@11.3), significant relative intensity changes and spectral shifts occur upon oxidation. The main carbonyl/carboxylate band, initially at 1626–1627 cm^–1^, decreases in intensity and shifts toward higher wavenumbers, reaching 1640 cm^–1^ for the lowest Fe­(II) density (Mh@1.1) and 1632–1633 cm^–1^ for higher densities. Concurrently, the shoulder at ∼1606 cm^–1^ upshifts to 1609–1611 cm^–1^ (Table 1).

This specific spectral signature mirrors our recent findings on the heterogeneous Fenton degradation of NAL triggered by hydrogen peroxide (H_2_O_2_).^44^ In this previous study, we established that the decoupling of the keto-carbonyl and carboxylate modes, manifested by the appearance of a band around 1635–1642 cm^–1^, is a fingerprint of the oxidative opening of the pyridinic ring, leading to a keto-carbonyl conjugated with a C=C bond.^57^ The fact that dark oxygenation (O_2_-driven) produces the same vibrational shifts as the classical Fenton reaction (H_2_O_2_-driven) implies that the reactive species generated by the simple aeration of sorbed Fe­(II) are capable of driving the same deep oxidation mechanisms, attacking the electron-rich C_3_ position (see Figure S7) and cleaving the aromatic core.

Furthermore, the evolution of the carboxylate band supports this ring-opening mechanism. Upon oxidation, the asymmetric carboxylate stretching mode shifts to approximately 1609–1611 cm^–1^. This position correlates well with vibrational frequencies reported for the nicotinate anion (1592–1614 cm^–1^),^58^ which shares a common structural core, a pyridine ring bearing a carboxylate group, with the ring-opened products. Indeed, the degradation byproducts identified by Ardo et al. (specifically, byproducts I and II; see Figure S7) consist of a substituted nicotinate moiety resulting from the cleavage of the quinolone N-containing ring.^3^ The emergence of this “nicotinate-like” spectral feature on the maghemite surface thus provides a direct structural link between the observed ATR-FTIR shifts and the chemical nature of the established oxidation products.

For the ferrihydrite series (Fh@0.2, Fh@5.6, and Fh@11.3), significant intensity changes are observed for the bands at 1624–1625 cm^–1^ and the shoulder at 1606–1608 cm^–1^ upon oxygenation, as observed in the Mh experiments (Figure 2). However, in contrast to the Mh series, the peak positions of these bands remain remarkably stable (Table 1). Even after 18 h of oxygenation, the main band remains centered at 1624–1625 cm^–1^, and the shoulder persists at 1606–1608 cm^–1^. Such a difference could imply that NAL degradation on Fh proceeds via a pathway preserving the quinolone ring (e.g., simple hydroxylation, similar to byproducts IV or V (see Figure S7) as described by Ardo et al.^3^). Alternatively, part of the sorbed nalidixate may be bound to ferrihydrite in an outer-sphere configuration, as discussed above. The final spectrum observed for the Fh series (Figure 2) is close to that of aqueous NAL,^45^ which could mask the signature of ring-opened byproducts.

Finally, to evaluate the overall molecular transformation kinetics, we monitored the absorbance at a fixed wavenumber, taken as the position of the main band at the end of the adsorption step (1626 cm^–1^ for Mh and 1624 cm^–1^ for Fh) throughout the entire experiment (Figure 3). The profiles clearly show the initial accumulation of NAL species on the Fe­(II)-loaded surfaces during the anoxic adsorption step (0–120 min). Upon introducing oxygen at t = 120 min, the signal first increases transiently in several of the systems before decaying. This initial increase is not observed in every experiment, and where it is observed, its amplitude bears no relation to the Fe­(II) surface loading; we attribute it to a perturbation of the laminar flow in the ATR measurement cell upon introduction of the oxidant. A sustained and progressive decay of the signal then follows for all systems over the subsequent 18 h. It should be noted that, for the maghemite series, the main band shifts from 1626 to 1632–1640 cm^–1^ during oxygenation (Table 1), so that the decrease measured at a fixed wavenumber reflects both the loss and the displacement of the band; on ferrihydrite, where the band position does not change, the two coincide. This confirms that the dark oxygenation of adsorbed Fe­(II) is capable of driving the transformation of antibiotic layers over time scales relevant to environmental redox cycles, regardless of the specific mineral topology.

**Figure 3:**
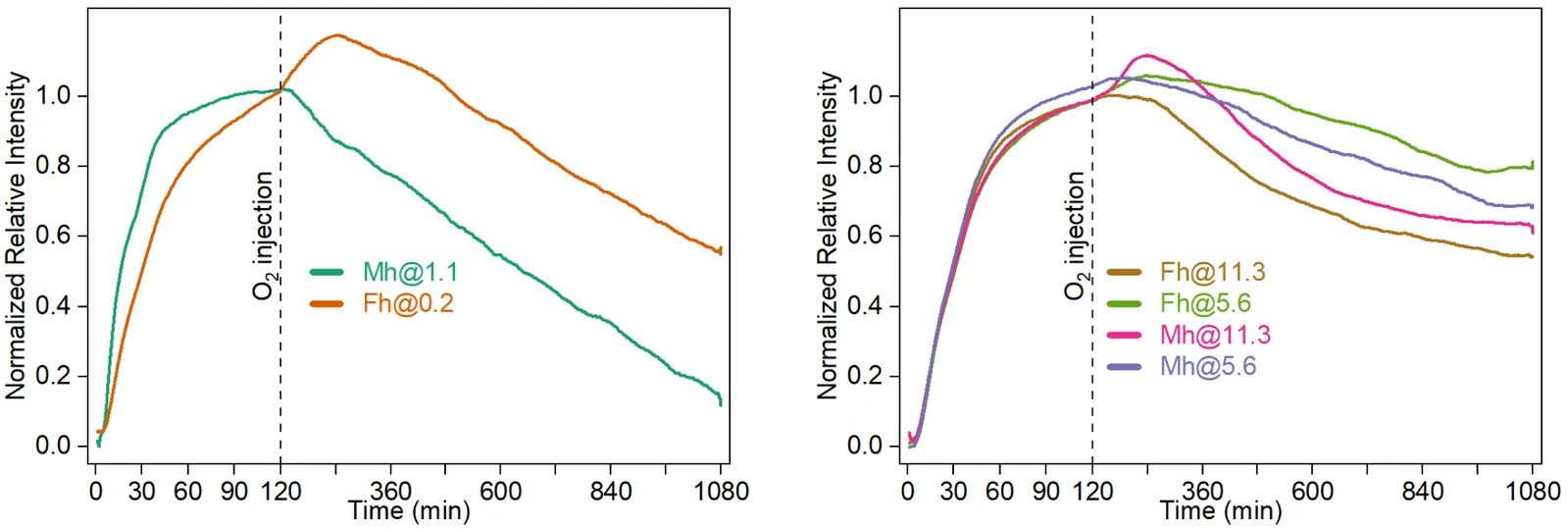
Temporal evolution of normalized peak absorbance intensity during nalidixate anion (NAL^–^) adsorption (0 to 120 min) and subsequent O_2_-induced degradation, shown separately for the first experimental series (left, Mh@1.1 and Fh@0.2, both at an initial nalidixate concentration of 0.026 mM in the recirculating system) and for the second series (right, Mh@5.6 and Mh@11.3 at 0.132 mM, Fh@5.6 and Fh@11.3 at 0.366 mM). Intensities were tracked at 1626 cm^–1^ for Mh and 1624 cm^–1^ for Fh, normalized to the value recorded at the end of the adsorption step (A/A_120_), and smoothed with a 20-point moving average. The vertical dashed line indicates O_2_ injection. Note that the time axis uses two different scales, 0 to 120 min and 120 to 1080 min, in order to display the adsorption and oxygenation regimes at comparable widths. The temporary increase in the signal observed for some experiments after the start of the O_2_ injection is due to a perturbation of the laminar flow in the measurement ATR cell.

## Environmental Implications

This study provides compelling *in situ* spectroscopic evidence that the “dark oxygenation” of Fe­(II) sorbed onto ubiquitous iron oxides is a potent pathway for the abiotic degradation of quinolone antibiotics. Crucially, the specific spectral signatures observed here under simple aeration mirror the ring-opening mechanism that we recently established for H_2_O_2_-driven heterogeneous Fenton systems.^44^ Here, we directly demonstrate that the reaction of sorbed Fe­(II) with dissolved oxygen generates reactive species powerful enough to induce deep structural changes in recalcitrant contaminants, without requiring strong oxidants such as hydrogen peroxide. Our Fe­(II)-sorbed maghemite experimental series directly reveals the presence of sorbed, ring-opened byproducts (“nicotinate-like” species), although these may be masked by remaining NAL^–^ outer-sphere complexes at the Fh surface; nevertheless, significant NAL^–^ degradation is observed on this mineral as well. These findings have significant implications for understanding the natural attenuation of emerging contaminants in redox-dynamic environments, such as wetlands, hyporheic zones, or aquifers subject to water table fluctuations. They suggest that Fe­(II)-bearing mineral phases act as sustainable reservoirs of reactivity, driving pollutant transformation over timescales of hours to days upon oxygen intrusion.

## Supplementary Material



## Notes

Complete time-resolved ATR-FTIR spectral data sets for the six systems, with the anoxic adsorption phase and the dark oxygenation phase shown separately (Figures S1 to S6); band positions of aqueous nalidixate compared with those of the sorbed species on both minerals (Table S1); molecular structures of nalidixic acid and its reported degradation products (Figure S7) (PDF)

## #

Laboratoire Génie des Procédés et Matériaux (LGPM), CentraleSupélec, Université Paris-Saclay, 8–10 rue Joliot-Curie, 91190 Gif-sur-Yvette, France
