# Supplementary material for: In Situ ATR-FTIR Analysis of Nalidixic Acid Degradation upon Oxygenation of Fe(II)-Sorbed Ferrihydrite and Maghemite Surfaces
Source: ACS Omega. 2026 Sep 24;11(39):60096–103. doi: 10.1021/acsomega.6c07545 (PMC13647406; doi:10.1021/acsomega.6c07545)
Supplement: Supplementary file 1 [file ao6c07545_si_001.pdf]

Supporting Information (SI)

In-situ ATR-FTIR analysis of nalidixic acid degradation upon oxygenation of Fe(II)-sorbed ferrihydrite and maghemite surfaces

Mbolantenaina Rakotomalala Robinson[1,2,3,†], Ana Carolina Schuh Frantz[1,3], Alberto Mezzetti[1], Sylvie Nélieu[4], Xavier Carrier[1,2], Guillaume Morin[3]*

[1]Sorbonne Université, CNRS, Laboratoire de Réactivité de Surface, LRS, F-75005 Paris, France

[2]Sorbonne Université, CNRS, Laboratoire Interfaces et Systèmes Electrochimiques, LISE, F-75005 Paris, France

[3]Institut de Minéralogie, de Physique des Matériaux et de Cosmochimie, IMPMC, UMR 7590, CNRS, Sorbonne Université, MNHN, 4 place Jussieu, 75252 Paris cedex 05, France

[4]Université Paris-Saclay, INRAE, AgroParisTech, UMR ECOSYS, 91120 Palaiseau, France

*Corresponding author. Email: mbolantenaina@yahoo.fr

[†]Present address: Laboratoire Génie des Procédés et Matériaux, LGPM, CentraleSupélec, Université Paris-Saclay, 8-10 rue Joliot Curie, 91190 Gif-sur-Yvette, France

Description of data presented: Table S1 compares the band positions of aqueous nalidixate with those of the sorbed species on both minerals. Figures S1 through S6 present the complete, time-resolved in-situ ATR-FTIR spectral datasets for the six experimental systems discussed in the main manuscript. Figure S7 depicts the molecular structures of nalidixic acid and its potential degradation products referenced in the discussion.

Table S1: Band positions (cm^{-1}) of aqueous nalidixate at pH 9, taken from Schuh Frantz et al.[1], compared with those of nalidixate sorbed on Fe(II)-loaded maghemite (Mh) and ferrihydrite (Fh) at the end of the adsorption step (t = 120 min). Δν(COO^-) is the difference between the asymmetric and symmetric carboxylate stretching positions and is considered as informative on the inner-sphere binding mode (Nakamoto 1986). Sorbed values are those of Table 1 of the main manuscript.

**Vibrational mode assignment**	**NAL^-(aq) pH 9**	**Mh@1.1**	**Mh@5.6**	**Mh@11.3**	**Fh@0.2**	**Fh@5.6**	**Fh@11.3**
νC=O carbonyl, νCOO^- as	1623	1627	1626	1626	1625	1624	1624
νC=O carbonyl, νCOO^- as	1609	1607	1606	1606	1607	1608	1606
νC–C 1st ring, νC–H	1574	1568	1570	1571	1571	1570	1572
νC–C and νC=N 2nd ring, δC–H	1525	1528	1522	1522	1523	1525	1522
νC–C and νC=N rings	1501	1502	1499	1499	1499	1501	1500
δC–H ethyl	1449	1451	1450	1449	1449	1449	1449
νCOO^- s, νC–C	1261	1262	1262	1262	1262	1262	1262
Δν(COO^-) = νCOO^- as – νCOO^- s	362	365	364	364	363	362	362

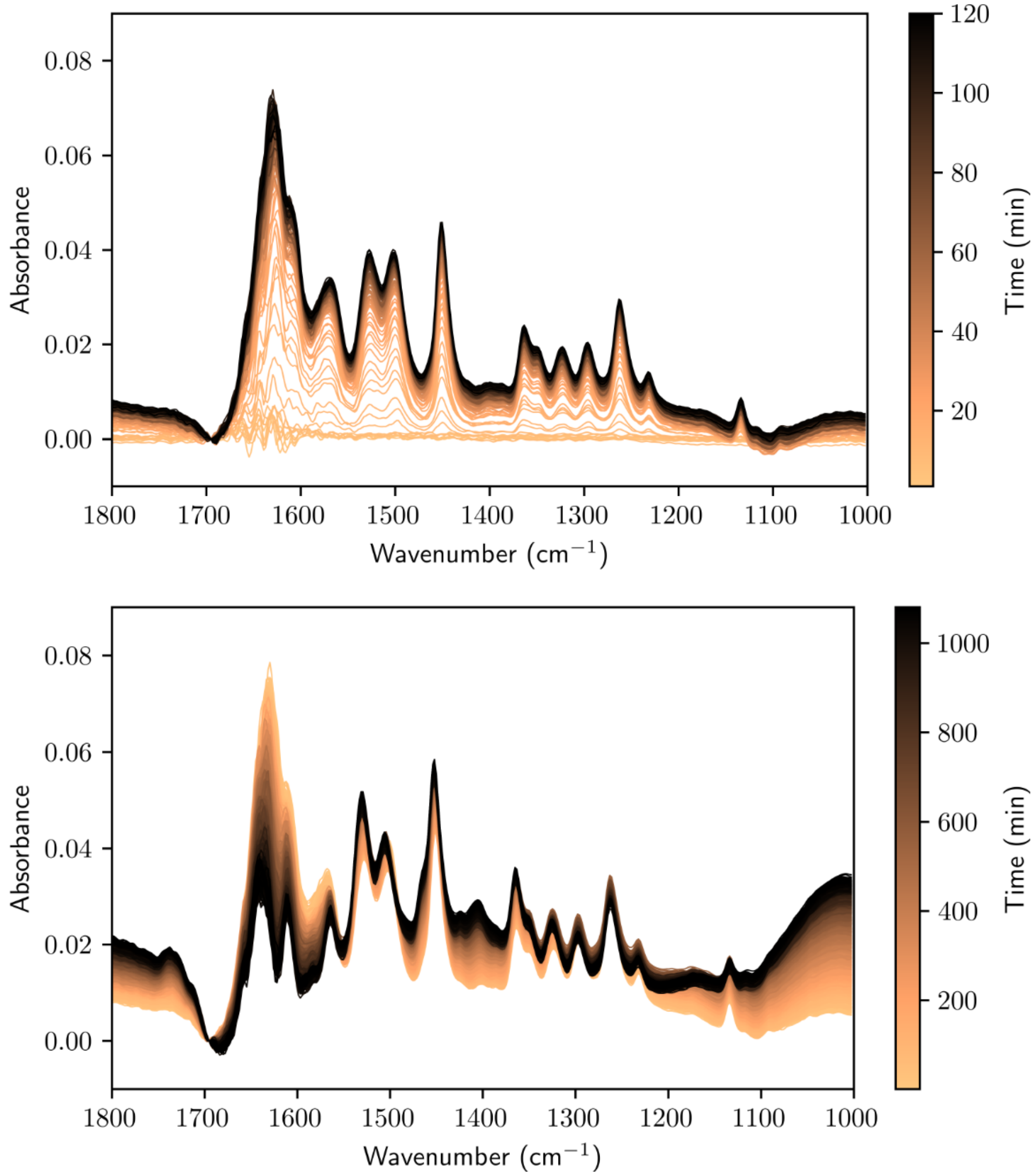

Figure S1: Complete time-resolved ATR-FTIR spectral evolution for the Mh@1.1 system (Maghemite, 1.1 at·nm^{-2} Fe(II)). (Top Panel) Anoxic adsorption phase: spectra acquired every minute during the 120 min equilibration with NAL^{-}. (Bottom Panel) Dark oxygenation phase: spectra acquired following the injection of O_2 at t=120 min (set as t=0 for degradation) and monitored for 18 hours.

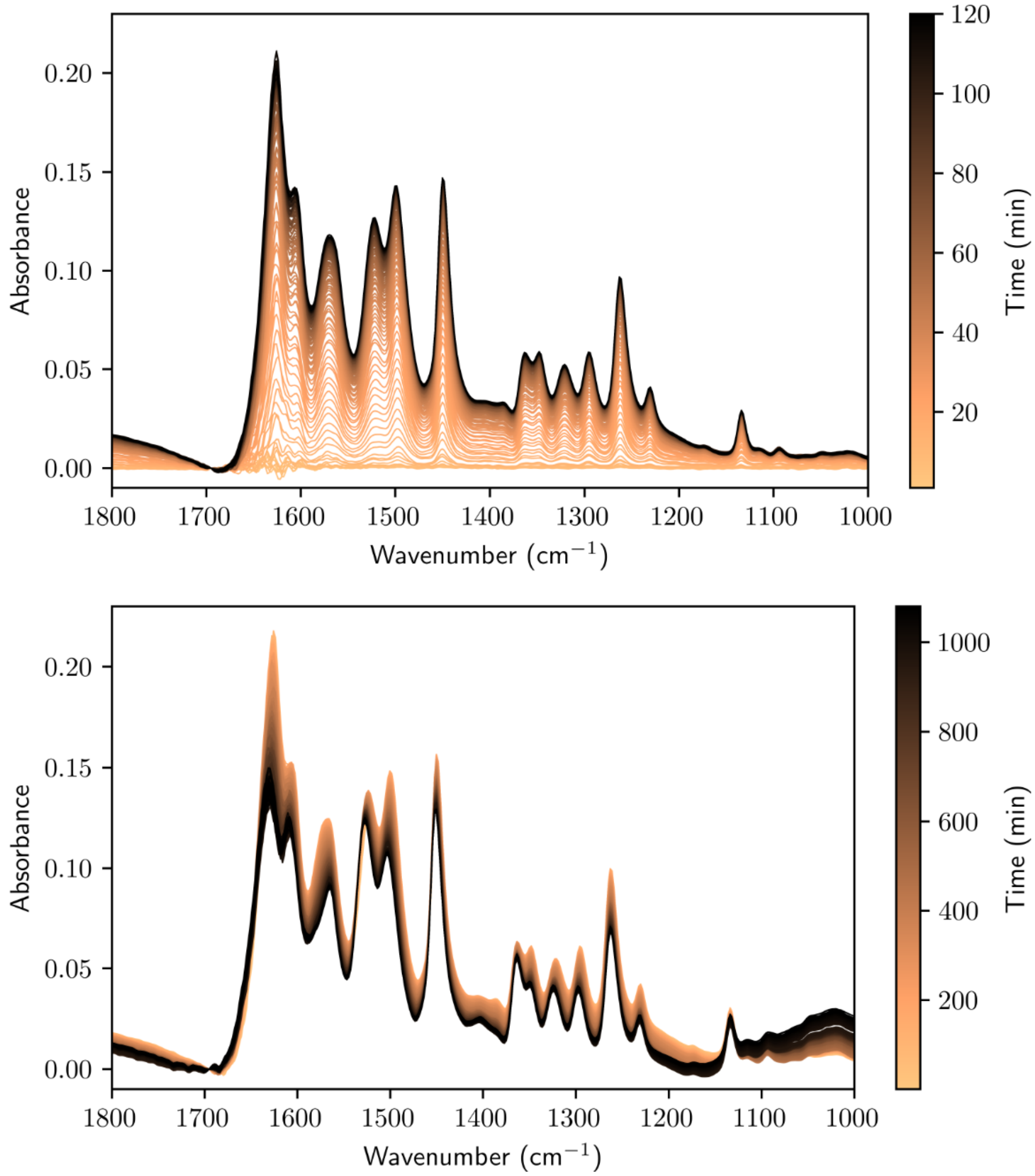

Figure S2: Complete time-resolved ATR-FTIR spectral evolution for the Mh@5.6 system (Maghemite, 5.6 at·nm^{-2} Fe(II)). (Top Panel) Anoxic adsorption phase: spectra acquired every minute during the 120 min equilibration with NAL^{-}. (Bottom Panel) Dark oxygenation phase: spectra acquired following the injection of O_2 at t=120 min (set as t=0 for degradation) and monitored for 18 hours.

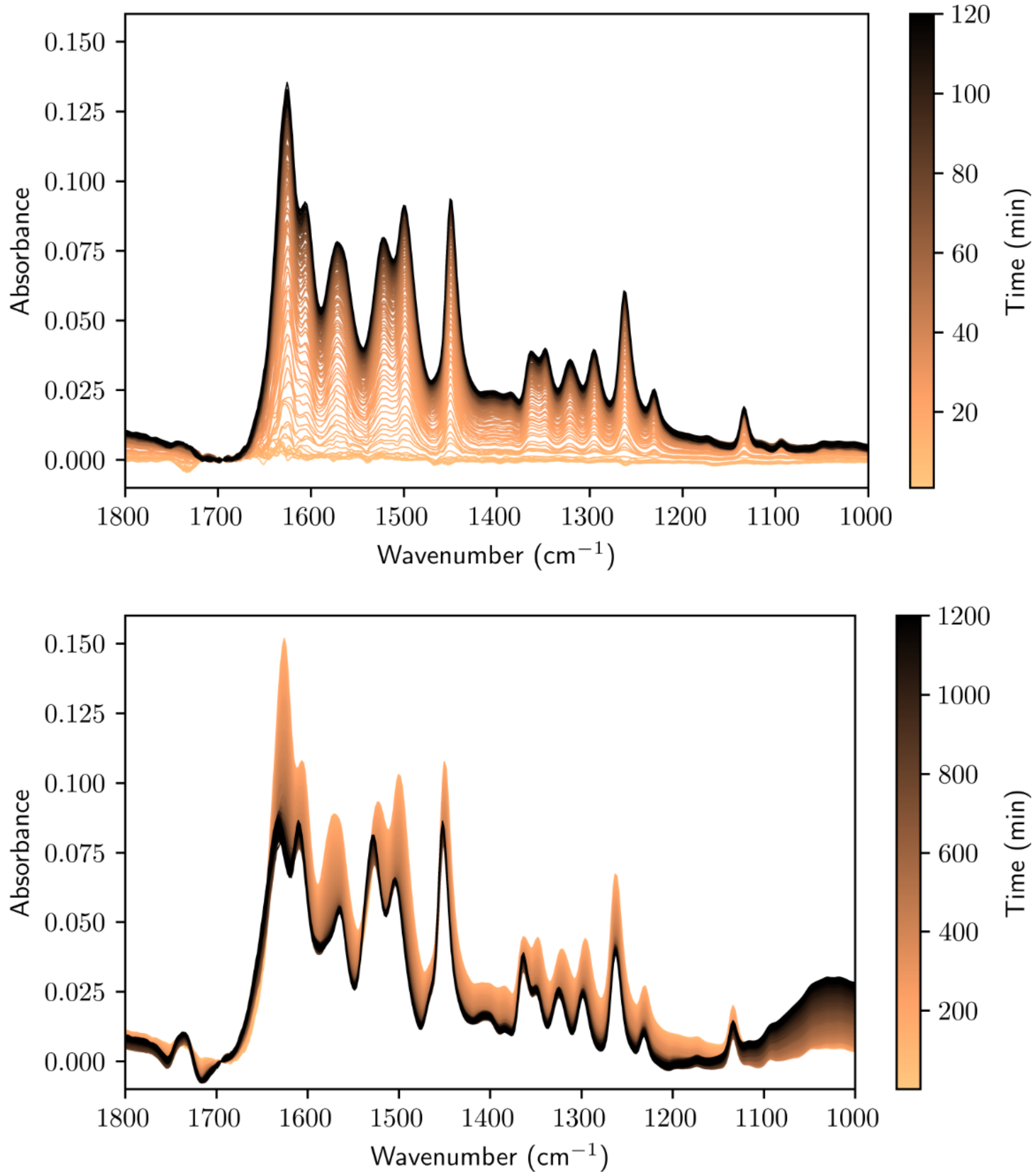

Figure S3: Complete time-resolved ATR-FTIR spectral evolution for the Mh@11.3 system (Maghemite, 11.3 at·nm^{-2} Fe(II)). (Top Panel) Anoxic adsorption phase: spectra acquired every minute during the 120 min equilibration with NAL^{-}. (Bottom Panel) Dark oxygenation phase: spectra acquired following the injection of O_2 at t=120 min (set as t=0 for degradation) and monitored for 18 hours.

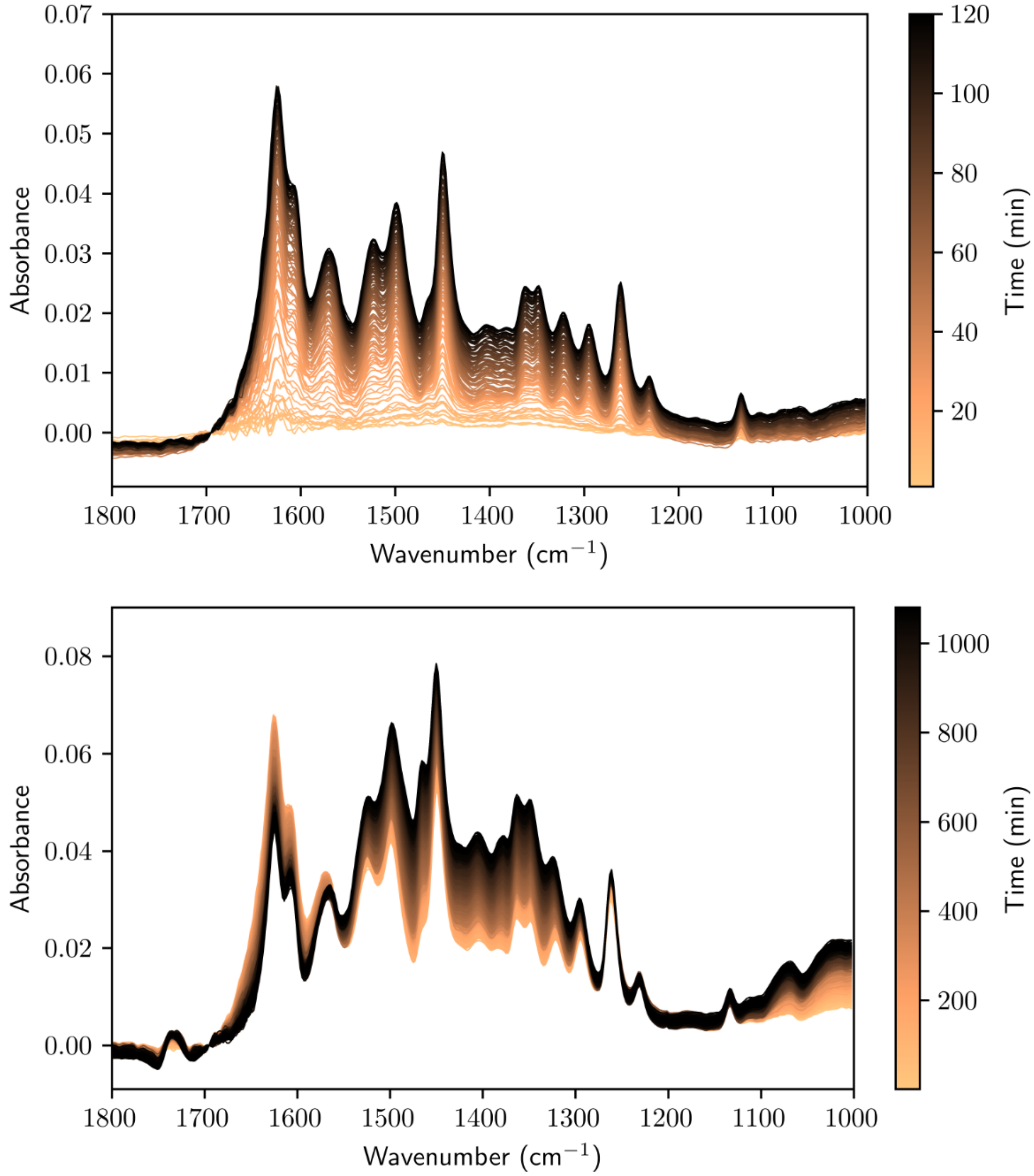

Figure S4: Complete time-resolved ATR-FTIR spectral evolution for the Fh@0.2 system (Ferrihydrite, 0.2 at·nm^{-2} Fe(II)). (Top Panel) Anoxic adsorption phase: spectra acquired every minute during the 120 min equilibration with NAL^{-}. (Bottom Panel) Dark oxygenation phase: spectra acquired following the injection of O_2 at t=120 min (set as t=0 for degradation) and monitored for 18 hours.

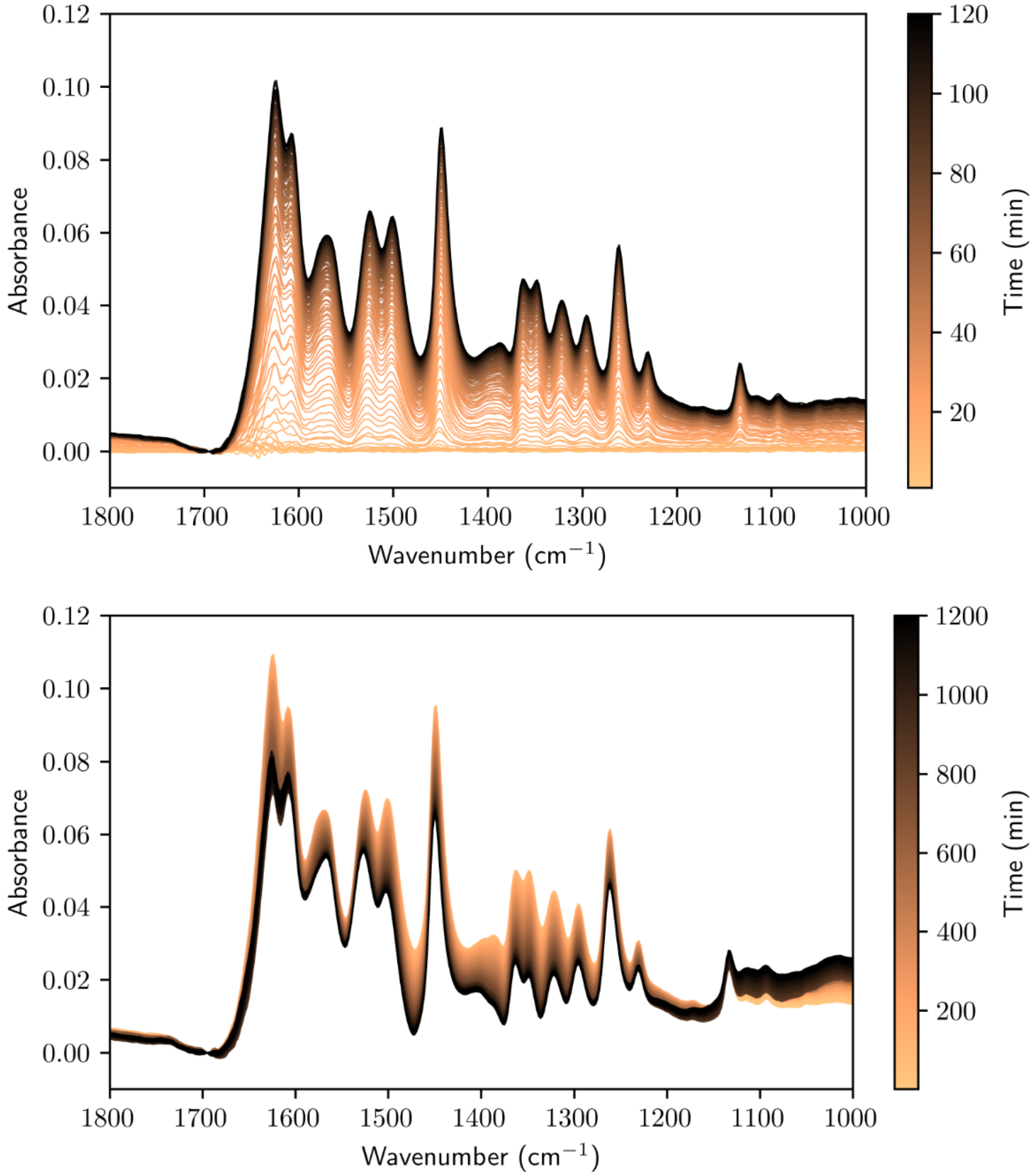

Figure S5: Complete time-resolved ATR-FTIR spectral evolution for the Fh@5.6 system (Ferrihydrite, 5.6 at·nm^{-2} Fe(II)). (Top Panel) Anoxic adsorption phase: spectra acquired every minute during the 120 min equilibration with NAL^{-}. (Bottom Panel) Dark oxygenation phase: spectra acquired following the injection of O_2 at t=120 min (set as t=0 for degradation) and monitored for 18 hours.

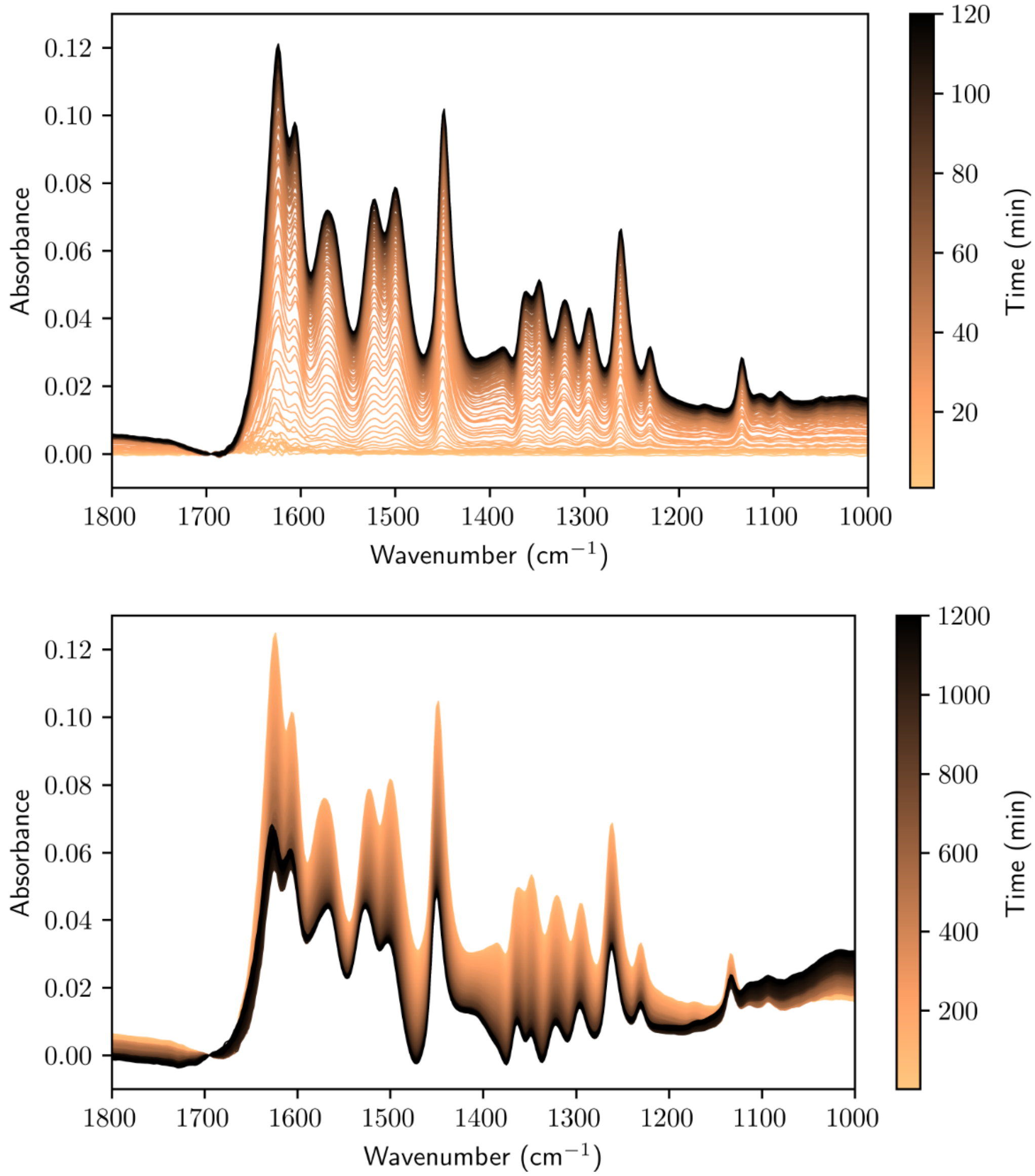

Figure S6: Complete time-resolved ATR-FTIR spectral evolution for the Fh@11.3 system (Ferrihydrite, 11.3 at·nm^{-2} Fe(II)). (Top Panel) Anoxic adsorption phase: spectra acquired every minute during the 120 min equilibration with NAL^{-}. (Bottom Panel) Dark oxygenation phase: spectra acquired following the injection of O_2 at t=120 min (set as t=0 for degradation) and monitored for 18 hours.

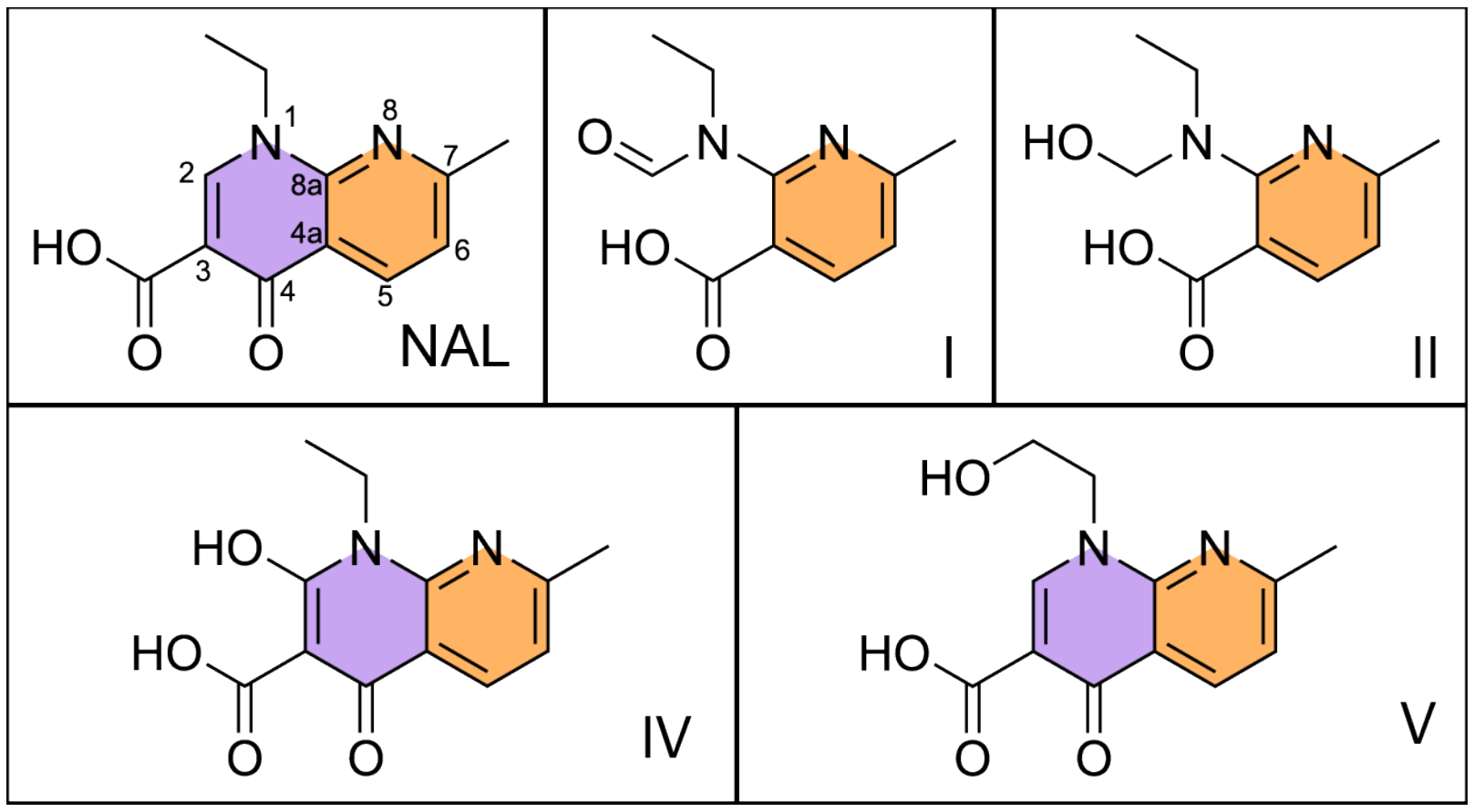

Figure S7: Molecular structures of nalidixic acid (NAL) and its reported degradation products. By-products I and II correspond to ring-opened species (nicotinate-like). By-products IV and V correspond to hydroxylated quinolone species[2].

References:

(1) Schuh Frantz, A. C.; Mezzetti, A.; Seitsonen, A. P.; Nélieu, S.; Balan, E.; Morin, G.; Carrier, X. Investigating Nalidixic Acid Adsorption onto Ferrihydrite and Maghemite Surfaces: Molecular-Level Insights via Continuous-Flow ATR-FTIR Spectroscopy. New J. Chem. 2024, 48 (46), 19403–19417. https://doi.org/10.1039/D4NJ03440F.

(2) Nakamoto K. Infrared and Raman Spectra of Inorganic and Coordination Compounds, Part B: Applications in Coordination, Organometallic, and Bioinorganic Chemistry, 4th ed.; John Wiley & Sons, 1986.

(3) Ardo, S. G.; Nélieu, S.; Ona-Nguema, G.; Delarue, G.; Brest, J.; Pironin, E.; Morin, G. Oxidative Degradation of Nalidixic Acid by Nano-Magnetite via Fe^{2+} /O_2 -Mediated Reactions. Environ. Sci. Technol. 2015, 49 (7), 4506–4514. https://doi.org/10.1021/es505649d.
